# Targeted or whole genome sequencing of formalin fixed tissue samples: potential applications in cancer genomics

**DOI:** 10.18632/oncotarget.4671

**Published:** 2015-07-31

**Authors:** Sarah Munchel, Yen Hoang, Yue Zhao, Joseph Cottrell, Brandy Klotzle, Andrew K. Godwin, Devin Koestler, Peter Beyerlein, Jian-Bing Fan, Marina Bibikova, Jeremy Chien

**Affiliations:** ^1^ Illumina, Inc., San Diego, CA, USA; ^2^ Department of Bioinformatics and Biosystems Technology, University of Applied Sciences Wildau, Wildau, Germany; ^3^ Department of Pathology and Laboratory Medicine, University of Kansas Medical Center, Kansas City, KS, USA; ^4^ Department of Biostatistics, University of Kansas Medical Center, Kansas City, KS, USA; ^5^ Department of Cancer Biology, University of Kansas Medical Center, Kansas City, KS, USA

**Keywords:** cancer genomics, FFPE DNA, whole exome sequencing, whole genome sequencing, copy number alterations

## Abstract

Current genomic studies are limited by the poor availability of fresh-frozen tissue samples. Although formalin-fixed diagnostic samples are in abundance, they are seldom used in current genomic studies because of the concern of formalin-fixation artifacts. Better characterization of these artifacts will allow the use of archived clinical specimens in translational and clinical research studies. To provide a systematic analysis of formalin-fixation artifacts on Illumina sequencing, we generated 26 DNA sequencing data sets from 13 pairs of matched formalin-fixed paraffin-embedded (FFPE) and fresh-frozen (FF) tissue samples. The results indicate high rate of concordant calls between matched FF/FFPE pairs at reference and variant positions in three commonly used sequencing approaches (whole genome, whole exome, and targeted exon sequencing). Global mismatch rates and C·G > T·A substitutions were comparable between matched FF/FFPE samples, and discordant rates were low (<0.26%) in all samples. Finally, low-pass whole genome sequencing produces similar pattern of copy number alterations between FF/FFPE pairs. The results from our studies suggest the potential use of diagnostic FFPE samples for cancer genomic studies to characterize and catalog variations in cancer genomes.

## INTRODUCTION

Detailed characterization of the genomic abnormalities in cancer cells is critical for our understanding and treatment of the disease [1]. Massively parallel sequencing technology has revolutionized this process, and it is now feasible to sequence entire cancer genomes for large numbers of samples in a timely and cost-efficient manner [2]. Comprehensive analysis of cancer genomes through whole genome, whole exome, and whole transcriptome approaches is revolutionizing our understanding of the types of somatic mutations and genomic rearrangements that can occur [3]. Characterization of these genomic changes across many cancer types and throughout different stages of the disease will be paramount for the development of novel therapies and will provide the foundation for personalized cancer treatment.

Cataloguing genomic alterations from large patient cohorts is critical for identifying true driver mutations from background mutation rates [4, 5]. Studies comparing DNA extracted from tumor and normal tissue have successfully identified somatic mutations across a number of different cancers by relying on sample sets of fresh-frozen (FF) tissues [6–11]. A major challenge in these types of studies is obtaining large numbers of fresh tissue samples that also have clinical information on disease progression and outcome [12]. Formalin fixation and paraffin embedding (FFPE) has been the standard sample preparation method for pathologists for decades, thus offering a vast resource of matched disease and normal tissues with clinically annotated samples and patient follow-up data [13]. The ability to sequence these large archives of FFPE samples would allow for powerful retrospective studies to investigate the complex genetic changes underlying progression of tumors, resistance to therapy, and variability in disease outcome.

Performing sequencing analyses using DNA isolated from FFPE samples is technically challenging. DNA extracted from FFPE blocks is highly variable due to DNA damage introduced by the fixation process. Formalin fixation causes hydrolysis of phosphodiester bonds, leading to varying degrees of DNA fragmentation [14]. Formalin crosslinking with cytosine nucleotides on either strand can result in incorrect incorporation of adenine in place of guanosine, causing an artificial C > T or G > A mutation [14, 15]. Despite the range of DNA quality found in the FFPE samples, previous studies have successfully used FFPE DNA for copy number analysis and mutation detection using targeted sequencing of single genes [16, 17], as well as the whole exome [18–20] and whole genome [21, 22]. While FFPE samples have a higher rate of non-reproducible sequence alterations, their random distribution allows for increases in coverage to reduce the false positive rate [19], thus making targeted sequencing a highly desirable approach for FFPE somatic mutation detection. Additionally, bioinformatics and statistical approaches are now being developed to deal with the background mutations present in FFPE samples [22]. These approaches will be essential for eliminating false-positive calls and improving sensitivity.

An additional challenge to working with FFPE samples is that DNA extracted from these tissues is often of limited quantity. While FFPE extraction methods have significantly improved [12, 23], DNA yields from these tissue types are often insufficient for standard next generation sequencing protocols [23]. Starting with low input amounts results in low diversity and poor uniformity sequencing data and ultimately severely inhibits the power of mutation detection [19]. Despite this limitation, several studies have sequenced samples starting with inputs as low as 10 ng. Whole genome analysis from 10 ng FFPE DNA input has been done, but successful analysis was limited to only changes in copy number [24]. An alternative approach for low inputs is targeted multiplex PCR [25]; however, this approach is not conducive to *de novo* mutation detection.

In this study, we adapted Illumina's transposase-based Nextera library preparation to generate high quality sequencing libraries from only 50 ng of starting genomic DNA. From this limited starting input, we successfully generated DNA sequencing libraries from 13 pairs of FF and FFPE matched samples that ranged in age and quality. The libraries can be used directly for whole genome sequencing (WGS) or further enriched for whole exome sequencing (WXS) or targeted exon sequencing (TES) of over 200 cancer-related genes. Our study provides a useful set of data from matched FF/FFPE pairs for whole genome (*n* = 2), low-pass whole genome (*n* = 14), whole exome (*n* = 26), and targeted exon sequencing (*n* = 26), and discusses and addresses potential artifacts from FFPE DNA sequencing.

## RESULTS

### Whole genome, whole exome, targeted exon and low-pass whole genome sequencing

**Figure 1:**
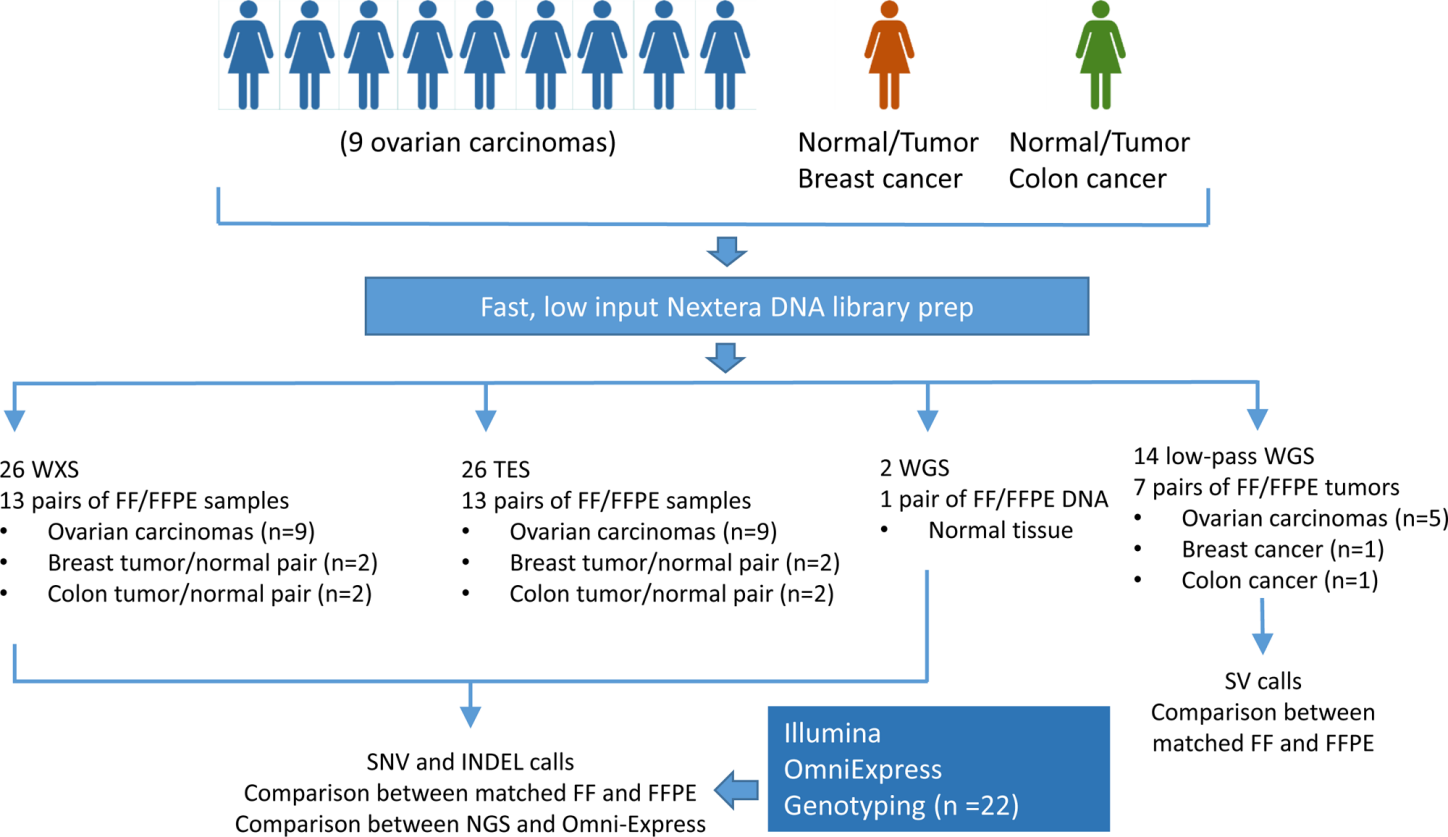
Study design and datasets To characterize sequencing artifacts in formalin-fixed, archived diagnostic samples, 13 pairs of patient-matched fresh frozen and formalin-fixed tissue samples were subjected to four popular sequencing approaches: whole exome sequencing (WXS), targeted exon sequencing (TES), whole genome sequencing (WGS), and low-pass whole genome sequencing. In addition, OmniExpress genotype array was used as an orthogonal platform to validate genotype calls.

To analyze the potential sequencing artifacts associated with DNA sequencing from formalin-fixed, paraffin-embedded (FFPE) clinical archived specimens, we generated four complementary DNA sequencing datasets from 13 pairs of fresh frozen (FF) and FFPE tissue samples (Table S1) and performed extensive characterization of DNA base calls, single nucleotide variation (SNV) calls, small insertions and deletions (INDELs), and copy number alterations (Figure 1). The data sets include 2 whole genome sequencing (WGS), 26 whole exome sequencing (WXS), 26 targeted exon sequencing (TES), and 14 low-pass whole genome sequencing (LP-WGS). Overall error rates and discordant base calls were used to characterize the contribution of base call quality, mapping quality, coverage, and minor allele frequency on discordant calls between FF and FFPE samples.

### Comparison of base calls between FF and FFPE samples

**Figure 2:**
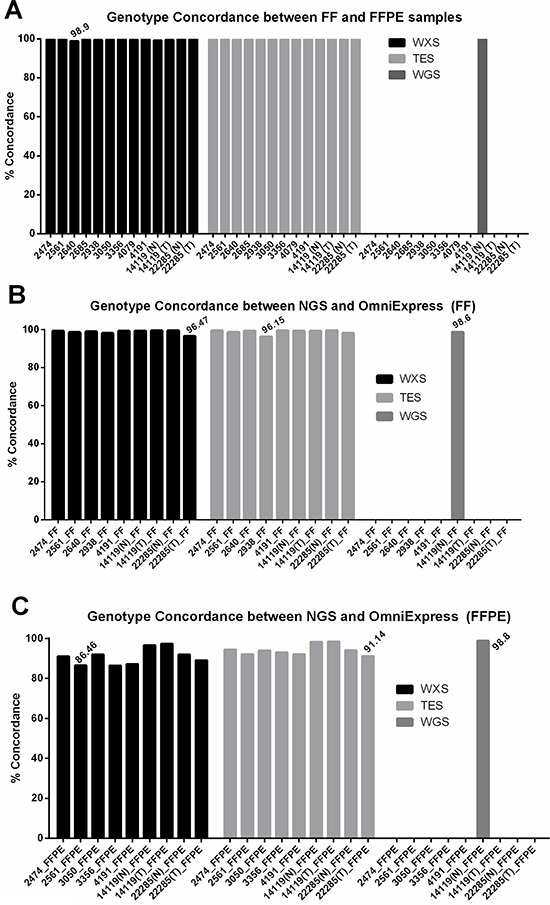
Concordance of base calls between matched FF and FFPE samples **A.** Concordance of base calls between FF and FFPE is higher than 98% in all samples in all three data sets (targeted exon sequencing, TES; whole exome sequencing, WXS, and whole genome sequencing, WGS). **B-C.** Concordance of base calls between Illumina sequencing and OmiExpress array-based genotyping is higher than 96% in all FF samples and 86% in all FFPE samples. In this analysis, both reference and alternate alleles were evaluated.

Since whole exome and targeted exon sequencing approaches are routinely used to assess sequence variations in the coding region of the genomes, we focused our analysis on 13 pairs of matched FF/FFPE DNA sequencing data sets. To assess the reliability of base calls from FFPE samples, we used consensus calls from the FF samples as the reference and classified FFPE base calls as concordant (same base call as in FF at the same position) or discordant (different base call compared to FF at the same position). Concordance rates of >98.9% (WXS) and >99.7% (TES) were observed within the paired samples (Figure 2A). Similarly, concordance of base calls between FF and FFPE in WGS data sets was 99.87% (Figure 2A). Detailed information on base calls and the definition of concordant and discordant base calls are provided in Table S2.

Illumina Omni Express array was used as an orthogonal platform for verification of base calls. Although base call concordance between SNP array and sequencing in FF and FFPE were comparable (Figure 2B and 2C, WGS), a higher rate of concordant calls was observed between sequencing and SNP array in FF samples than in matched FFPE samples in WXS and TES data sets (Figure 2B and 2C).

### Comparison of single nucleotide variant (SNV) calls between FF and FFPE samples

**Figure 3:**
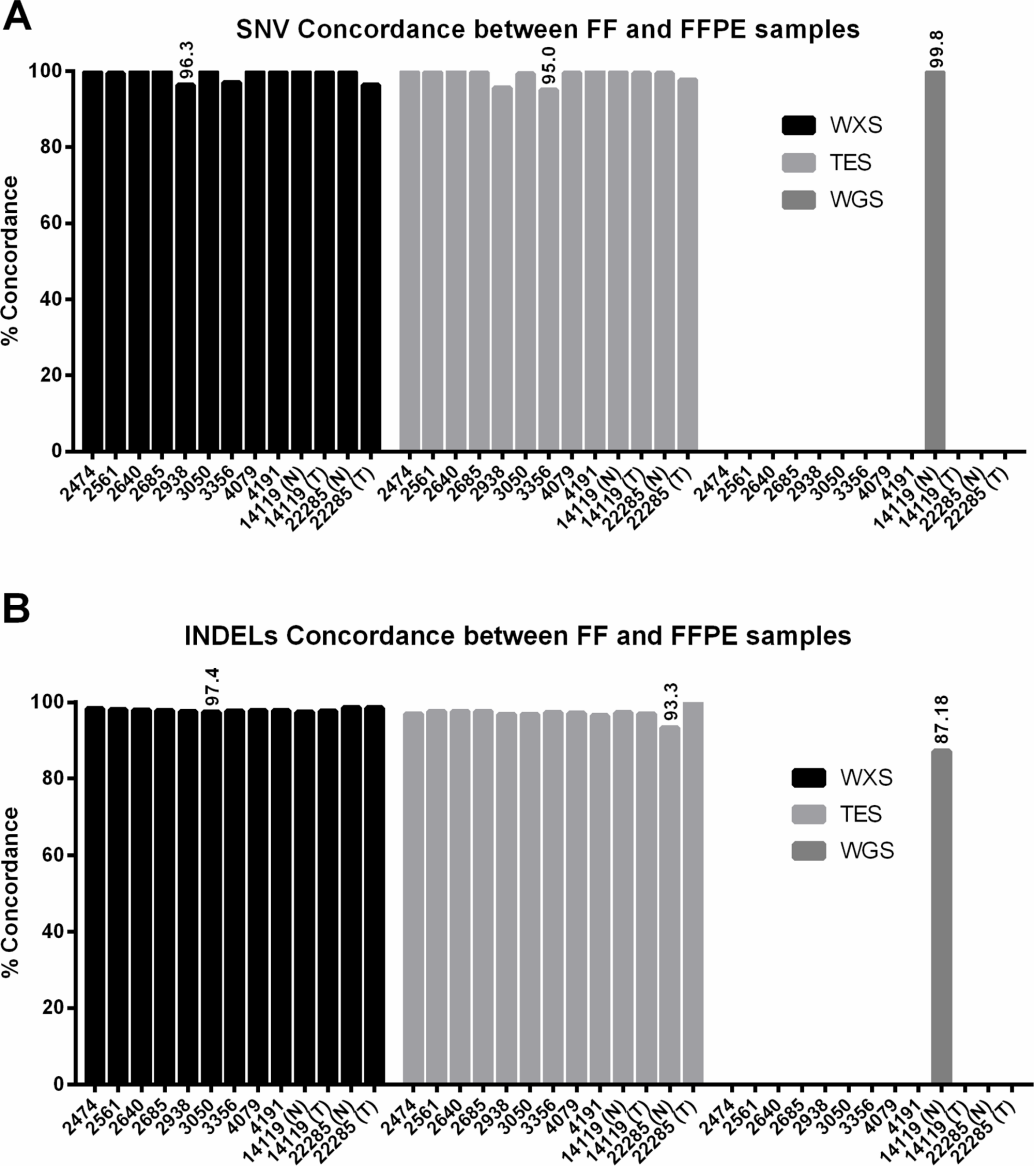
Concordance of single nucleotide variant calls and small insertion and deletion calls between matched FF and FFPE samples **A.** Concordance of SNV calls between FF and FFPE is higher than 95% in all three data sets. **B.** Concordance of INDEL calls between FF and FFPE is higher than 87% in all data sets.

Next, we compared SNV calls between FF and FFPE samples using GATK genotype caller [26]. We observed that >96% of SNVs in WXS from FFPE samples were concordant with those from corresponding FF samples when all overlapped positions were analyzed (Figure 3A and Table S3). Similarly, we observed that >95% of SNVs in TES and >99.8% of SNVs in WGS from FFPE samples were concordant with those in corresponding FF samples (Figure 3A and Table S3).

For INDEL calling, we also chose a threshold of 13X coverage and mapping quality of 43 for WGS and WXS. In TES we chose a threshold of 20X coverage and mapping quality of 43. Methods for INDEL detection and calling are not as mature as those for SNV calling, and technical issues with gapped alignment still are problematic and contribute to challenges associated with INDEL callers. Nevertheless, we found high levels of concordance in WXS (>97%), TES (>93%), and WGS (>87%) between FF and FFPE samples (Figure 3B and Table S4). For the INDEL calling, we observed a mean insertion size of 6 bp (range 1–165 bp) and a mean deletion size of 9 bp (range 1–126 bp) in FF samples and a mean insertion size of 5 bp (range 1–165 bp) and a mean deletion size of 8 bp (range 1–126 bp). The average coverage at these INDELs is 79X in FF samples and 61X in FFPE samples. Manual inspection of the putative false-positive and false-negative positions in Samtools pileup indicates missed calls by the INDEL caller in the majority cases, i.e., for false-positives that were called in FFPE samples but not in FF samples, manual inspection of the positions in FF indicates presence of INDELs (data not shown). Since INDEL callers are not as robust as SNV callers, such missed calls are expected.

Since some of the tumor samples had matched normal DNA, we also performed somatic mutation analysis between matched normal and tumor samples. We observed that approximately 1,000 positions out of 1.5 million targeted positions are variant in both normal and tumor samples (Table 1). Upon subtraction of germline variants, we observed an average of 42 somatic mutations in the samples in the TES data sets. In WXS data sets, we observed >6, 200 positions are variant in both normal and tumor samples. We also observe an average of approximately 447 somatic mutations in the samples in the WXS data sets. In sample 14119, we observed 387 and 491 putative somatic mutations in FF and FFPE samples, respectively (Table 1). Ninety somatic SNV positions show overlap between FF and FFPE samples, and within the overlapped positions, we observed 88 concordant calls and only two discordant calls for somatic mutations. These results point to the feasibility as well as challenges associated with generating somatic mutation calls from FFPE samples that are concordant with somatic mutation calls from FF samples. It should be noted that due to limitations in the INDEL caller and variations in coverage of any given region between matched FF and FFPE samples, INDEL caller is expected to miss some INDELs in FF (contributing to false-positive results) and in FFPE (contributing to false-negative results). However, our results show that when INDEL calls are made in both FF and FFPE samples, these calls are usually consistent.

**Table 1:** Somatic mutations

WXS	Normal	Tumor	Same positions	Concordant	Somatic	Same positions*	Concordant	Discordant
14119_FF	19750	19852	19024	19013	387			
14119_FP	19655	18807	18301	18252	491	90	88	2
22285_FF	15521	11253	10670	10472	461			
22285_FP	13920	10765	6296	6058	450	55	54	1
**TES**								
14119_FF	1274	1259	1189	1188	30			
14119_FP	1266	1214	1126	1124	47	6	5	1
22285_FF	2157	2052	1068	1063	42			
22285_FP	1219	881	416	408	49	5	5	0

SNVs in normal and tumor samples were identified in whole exome and targeted exon sequencing data sets. Overlap of positions between normal and tumor within FF or FP are indicated by “Same positions”. Overlap of positions between normal and tumor within FF and FP (all four samples) are indicated by “Same position*”. Among the overlap positions in all four samples, concordant and discordant somatic calls are shown next.

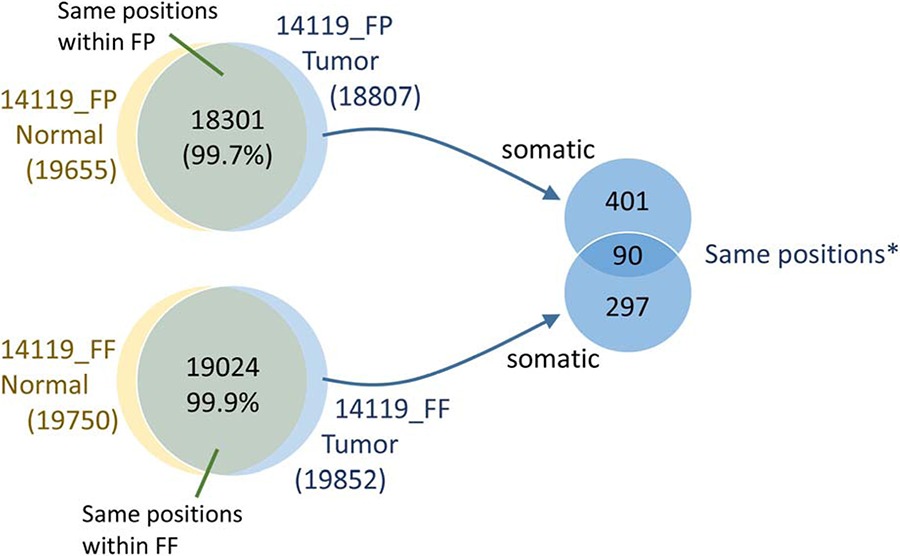

After filtering out synonymous SNVs and those present in normal samples (14119 and 22285) or in more than 10% of the 1000 Genome Project, we compiled a list of genes with SNVs detected in 2 or more FF/FFPE pairs from TES dataset (Table 2). SNVs that are reported in the Catalogue of Somatic Mutations in Cancer (COSMIC) database are indicated by COSMIC IDs. Some of the top candidates include MLL3 (8 out of 11 tumor samples) and TSC1 (6 out of 11) which are previously reported to be mutated in human carcinomas. Interestingly, T316S SNV in MLL3 is not detected in FF and FFPE normal sample (14119N) but detected in both FF and FFPE tumor sample (14119T). However, since the majority of tumor samples do not have matching normal samples, it is not clear if T316S SNV in MLL3 is tumor-specific.

**Table 2:** Variants found in at least 2 tumor samples from TES dataset

Count	Gene	AAChange	CosmicID	2474	2561	2640	2685	2938	3050	3356	4079	4191	14119 (N)	14119 (T)	22285 (N)	22285 (T)
8	NEB	NM_001164507:c.T2885G:p.V962G	COSM304166													
8	CNTNAP2	NM_014141:c.A1765C:p.T589P	COSM1284196													
8	MLL3	NM_170606:c.A946T:p.T316S														
7	CACNA2D1	NM_000722:c.T620G:p.V207G														
6	ADAMTS20	NM_025003:c.C540A:p.N180K														
6	GJB2	NM_004004:c.T500G:p.V167G														
6	TSC1	NM_001162427:c.T2752G:p.L918V														
6	UBAP2	NM_018449:c.A1487C:p.H496P														
6	IGFBP7	NM_001253835:c.C31T:p.L11F														
5	KCNJ12, KCNJ18	NM_001194958:c.G889A:p.V297I														
5	KCNJ12, KCNJ18	NM_001194958:c.G906T:p.M302I														
5	TACC3	NM_006342:c.G427A:p.E143K														
5	CNTNAP2	NM_014141:c.A3352C:p.T1118P	COSM1284198													
5	COL1A1	NM_000088:c.A3772C:p.T1258P														
5	DNAH7	NM_018897:c.A475G:p.K159E														
5	CTNNB1	NM_001098209:c.C1995A:p.D665E														
5	PTK2B	NM_173175:c.C2753G:p.A918G														
4	ATM	NM_000051:c.G5557A:p.D1853N	COSM41596													
4	KCNJ12, KCNJ18	NM_001194958:c.G865C:p.E289Q	COSM312202													
4	KCNJ12, KCNJ18	NM_001194958:c.C869T:p.T290M														
4	PKHD1L1	NM_177531:c.A490G:p.I164V														
4	XIRP2	NM_001199144:c.A3286C:p.T1096P														
4	XIRP2	NM_001199144:c.A1873C:p.T625P														
4	SULF2	NM_001161841:c.G226A:p.A76T	COSM1412272													
4	CHEK2	NM_007194:c.G736T:p.V246L	COSM304712													
4	TNK2	NM_001010938:c.A1601C:p.D534A														
4	NHS	NM_001136024:c.T113C:p.L38P	COSM1317240													
3	MGMT	NM_002412:c.A520G:p.I174V														
3	ERBB3	NM_001982:c.A3355T:p.S1119C														
3	BARD1	NM_000465:c.G1670C:p.C557S														
3	GABRA6	NM_000811:c.C1210T:p.P404S														
3	PKHD1	NM_138694:c.C1736T:p.T579M														
3	MLL3	NM_170606:c.G2512A:p.G838S														
3	MLL3	NM_170606:c.A2185G:p.N729D	COSM1635198													
3	MLL3	NM_170606:c.A14062C:p.T4688P														
3	ABCB1	NM_000927:c.A61G:p.N21D	COSM1178512													
3	PKHD1L1	NM_177531:c.C4403T:p.S1468F	COSM304040													
3	NOTCH1	NM_017617:c.A580C:p.T194P	COSM1624741													
3	PIK3CD	NM_005026:c.A1127G:p.E376G														
3	KIAA1549L	NM_012194:c.A3656C:p.N1219T														
3	ADAMTS20	NM_025003:c.T3137G:p.V1046G														
3	NOTCH3	NM_000435:c.A982C:p.T328P														
3	HJURP	NM_018410:c.G1643C:p.S548T														
3	ADAMTS2	NM_014244:c.G722A:p.R241H														
3	USP17L7	NM_001256869:c.G902T:p.R301L														
2	MKI67	NM_001145966:c.G7958T:p.R2653L														
2	MKI67	NM_001145966:c.A6656T:p.D2219V	COSM328282													
2	MKI67	NM_001145966:c.C4550T:p.P1517L														
2	MKI67	NM_001145966:c.G3595A:p.V1199M	COSM146354													
2	MKI67	NM_001145966:c.C2660T:p.T887I	COSM146356													
2	MKI67	NM_001145966:c.A811C:p.I271L	COSM146358													
2	ANKRD30A	NM_052997:c.C374T:p.T125M														
2	MUC2	NM_002457:c.C3620T:p.T1207I	COSM1351086													
2	PARP4	NM_006437:c.A3176G:p.Q1059R														
2	KCNJ12, KCNJ18	NM_001194958:c.G782A:p.R261H	COSM312197													
2	KCNJ12, KCNJ18	NM_001194958:c.T785G:p.I262S	COSM312198													
2	MUC4	NM_018406:c.C6671T:p.P2224L	COSM1644167													
2	MUC4	NM_018406:c.C5854T:p.P1952S	COSM1042915													
2	MUC4	NM_018406:c.G5271C:p.Q1757H	COSM149606													
2	MUC4	NM_018406:c.T5971C:p.S1991P	COSM1042911													
2	MAP3K1	NM_005921:c.C2816G:p.S939C														
2	PKHD1	NM_138694:c.T1756G:p.F586V														
2	MLL3	NM_170606:c.C2315T:p.S772L														
2	DNAH11	NM_003777:c.G7573A:p.V2525I														
2	DNAH11	NM_003777:c.C1961G:p.S654C														
2	DNAH11	NM_003777:c.T7777C:p.Y2593H														
2	PKHD1L1	NM_177531:c.T11416G:p.C3806G	COSM304038													
2	NOTCH1	NM_017617:c.C2734T:p.R912W														
2	NOTCH1	NM_017617:c.A931C:p.T311P														
2	ADAM12	NM_003474:c.G212A:p.R71Q														
2	FAT3	NM_001008781:c.C1235T:p.S412F														
2	RASAL1	NM_001193521:c.C173T:p.T58M														
2	HERC1	NM_003922:c.G3415T:p.V1139L														
2	HERC1	NM_003922:c.C9455T:p.S3152F														
2	XIRP2	NM_001199145:c.A1603G:p.R535G														
2	ABCC1	NM_004996:c.G2012T:p.G671V														
2	DNMT1	NM_001130823:c.G206A:p.R69H														
2	LRP1B	NM_018557:c.C4174T:p.L1392F														
2	LRP1B	NM_018557:c.T8707G:p.C2903G	COSM1631297													
2	PIKFYVE	NM_015040:c.A1849G:p.M617V														
2	PIKFYVE	NM_015040:c.T3097G:p.S1033A														
2	DSP	NM_001008844:c.A913T:p.I305F	COSM1685467													
2	FLNC	NM_001127487:c.G4700A:p.R1567Q														
2	CDKN2A	NM_000077:c.G442A:p.A148T														
2	GPR179	NM_001004334:c.C2650T:p.R884W														
2	ADAMTS2	NM_014244:c.G2480A:p.R827Q														
2	FLNA	NM_001456:c.A5747C:p.Y1916S														

Table legend: Samples with indicated variants are shown in red. Gray blocks represent absence of variants in the samples. Only variants found in matching FF/FFPE pair are shown here. Genes known to be highly variant (such as MUC16, MUC4, HLAs) or variants that are present in at least 10% of 1000 Genome Project or detected in normal samples (14119 or 22285) are filtered out. Variants are listed in the order from the highest to the lowest frequency in the dataset. It is important to note that not all variants listed here are expected to be somatic. Since normal matching samples were not available for the majority of samples (except samples 14119 and 22285), the list may include uncommon germline variants.

### Comparison of copy number variations (CNVs) between FF and FFPE samples

**Figure 4:**
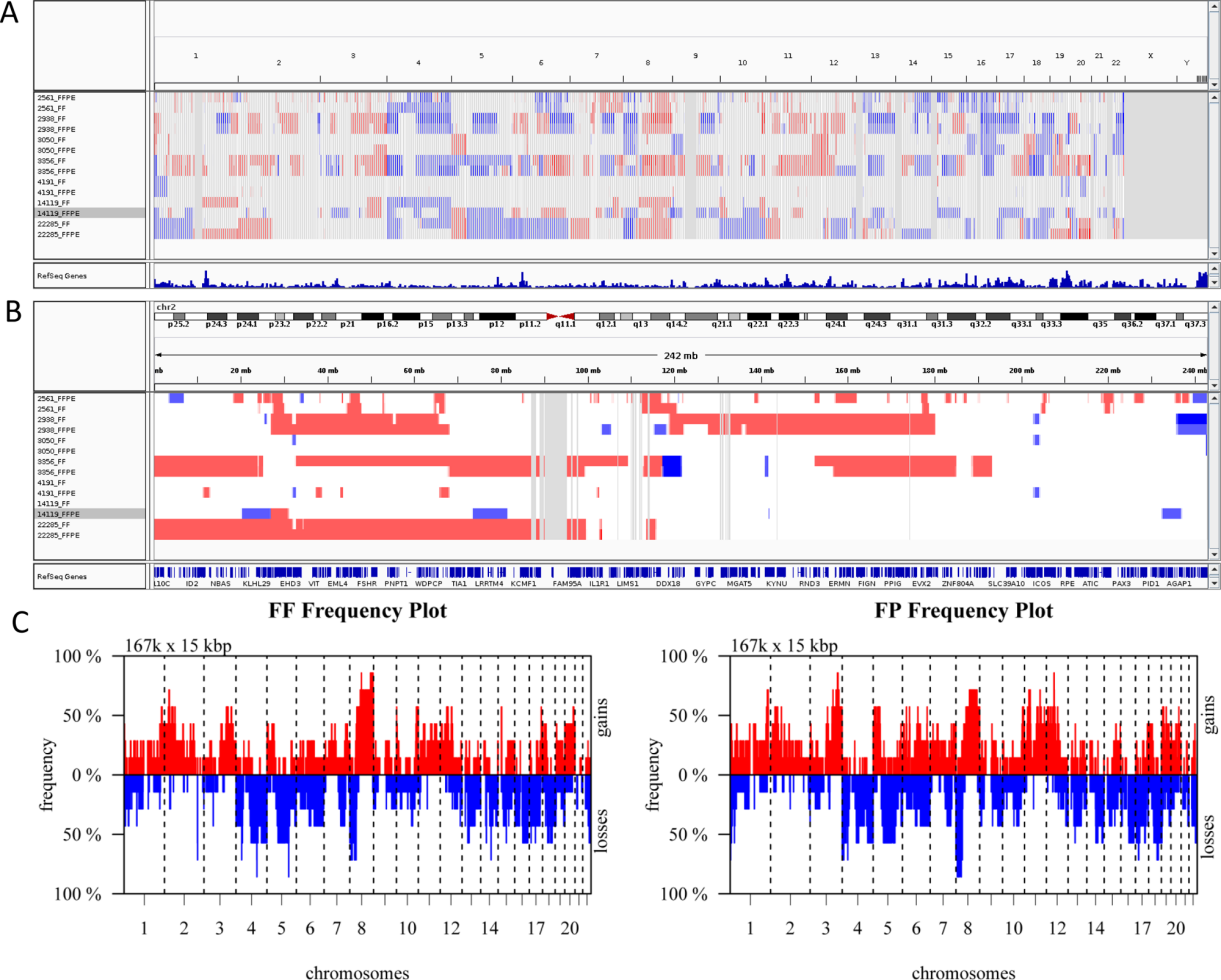
Analysis of Copy Number Variations (CNVs) in FF and FFPE tumor pairs Copy number variations in tumor samples were determined using QDNAseq and visualized by Integrative Genome Viewer. **A.** Whole genome view with copy number loss (blue) and copy number gain (red) regions are highlighted for all 7 pairs of tumor samples. **B.** Copy number variations in Chromosome 2 are shown for all 7 pairs of FF and FFPE samples. **C.** Copy number profiles of FF and FFPE (FP) tumor groups show similar pattern of gains and losses. Frequency of copy number alterations are plotted on Y-axis, and chromosome coordinates are plotted on X-axis and include chromosome 1 to 22. Plot was generated using CGHbase R package.

**Figure 5:**
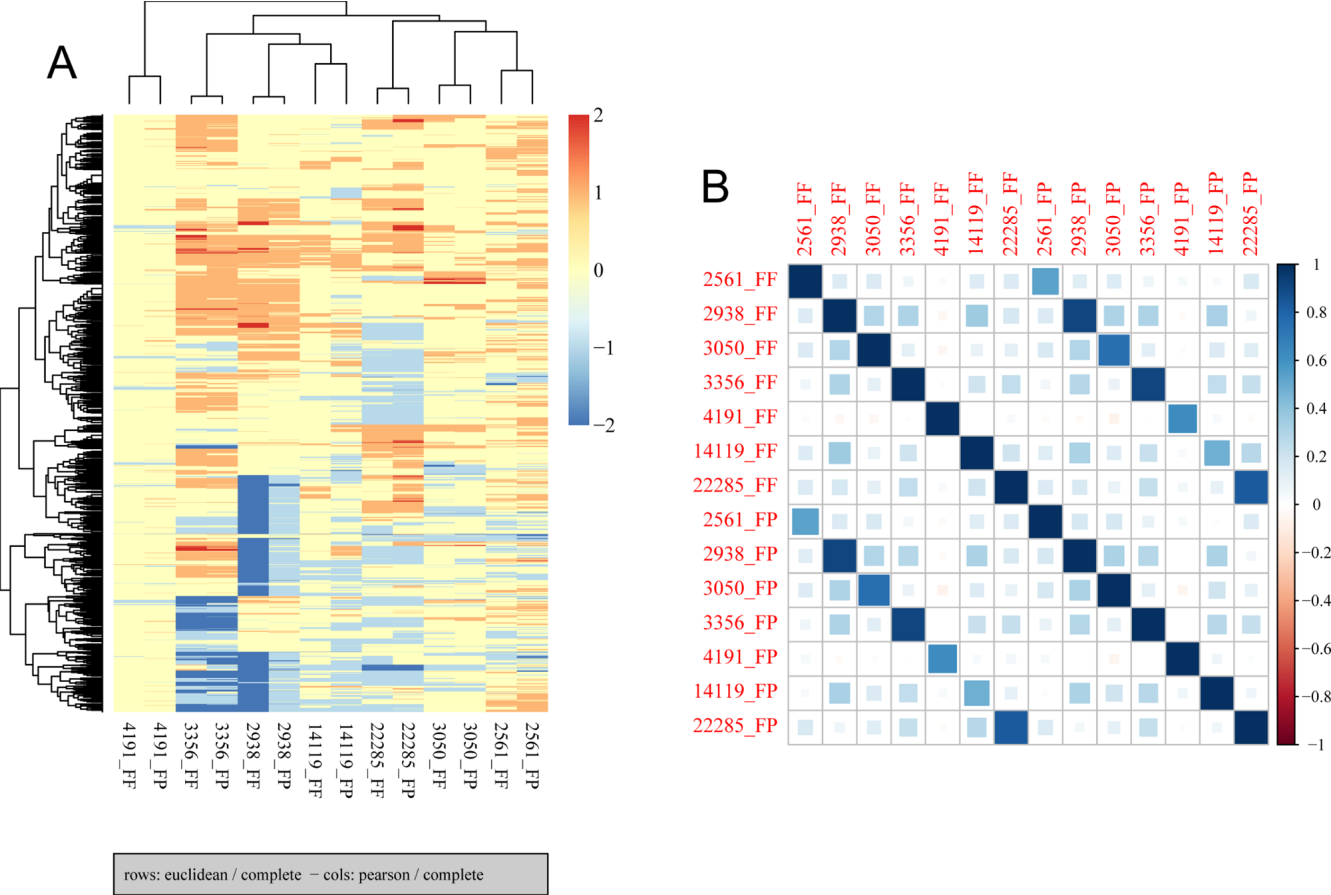
Hierarchical clustering and correlation analysis of copy number alterations in FF and FFPE samples **A.** Non-supervised hierarchical clustering was performed using aheatmap R package with input data created by CGHregions R package. The results show clustering of FF/FFPE pairs, indicating similarity between paired samples. **B.** Pearson correlation was performed to assess the correlation across all samples, and results indicate paired samples are highly correlated.

Finally, we compared CNVs detected in paired FF and FFPE samples using low-pass (0.2x mean coverage after duplicate removal) whole genome sequencing data generated from seven pairs of FF and FFPE tumor samples (Table S5). In general, we observed similar variations in mappability due to variations in GC content (Figure S1) and similar patterns of variation in coverage within segmented regions (Figure S2). Variations in copy numbers within segmented regions between paired FF and FFPE samples are similar although the size of predicted CNVs differed between paired samples (Figure 4). The median and the range in the size of CNVs are comparable between FF and FFPE groups (Table S6). Non-supervise hierarchical clustering of copy number alterations from these data sets indicates that FF and FFPE samples from the same patient clustered together (Figure 5A). Finally, Pearson correlation analysis showed that paired samples are strongly correlated (Figure 5B). These results demonstrate the feasibility of performing low-pass whole genome sequencing to detect putative copy number variations in DNA extracted from FFPE tumor samples.

### Characterization of known FFPE artifacts

**Figure 6:**
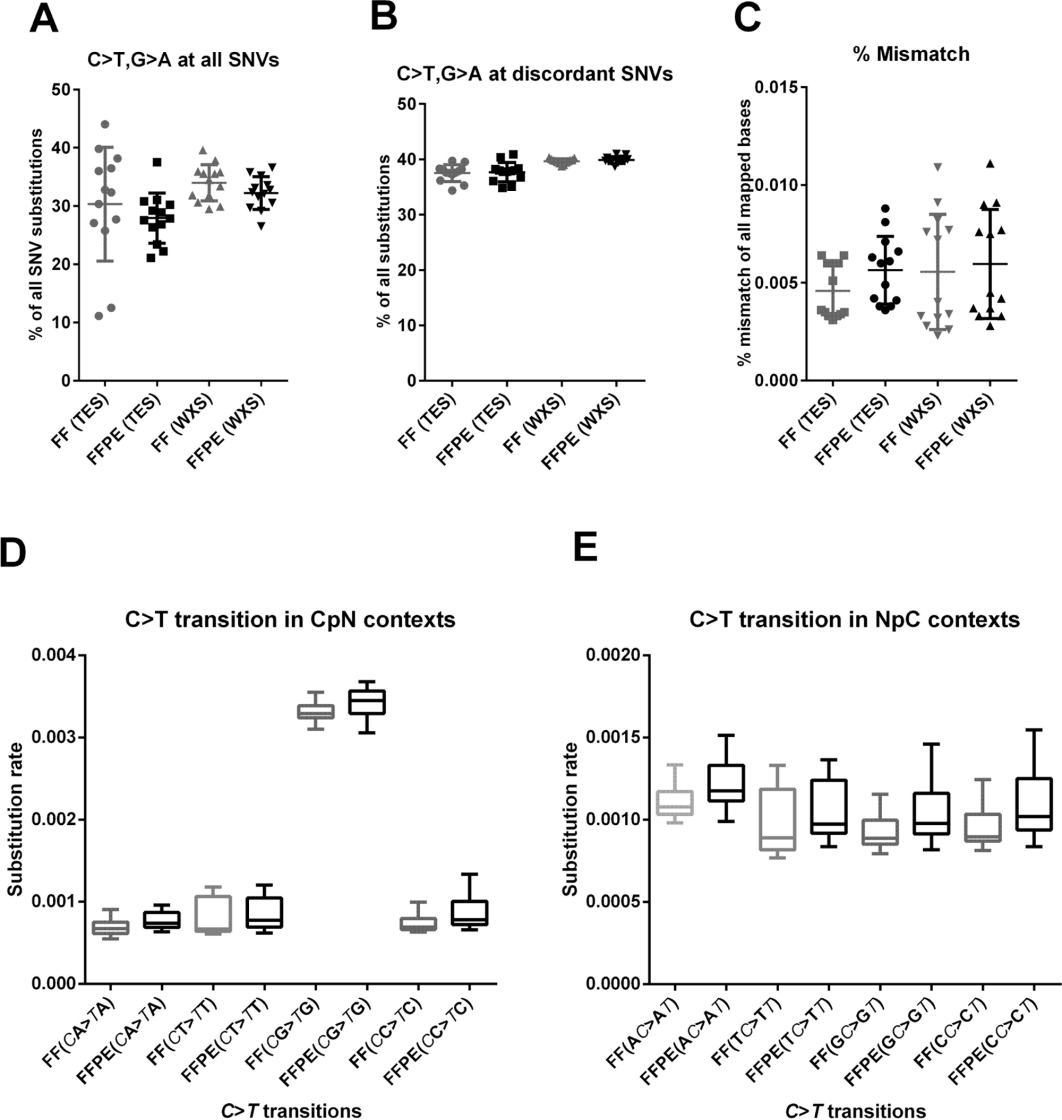
Characterization of FFPE artifacts **A.** Combined rates of C > T and G > A transition in TES and WXS data sets show no significant difference between FF and FFPE samples when all variant positions are analyzed (*P* = 0.4872 and *P* = 0.1845, respectively). **B.** In contrast, C > T or G > A (in reverse strand) substitution rates at discordant positions is marginally higher in FFPE samples in WXS data sets (*P* = 0.0201) but not in TES data sets (*P* = 0.2531). **C.** Global mismatch rates are slightly higher in FFPE samples compared to FF samples in both TES and WXS data sets, but they are not significant (*P* = 0.0704) in TES data sets and significant in WXS data sets (*P* = 0.0392). **D.** C > T substitutions are substantially higher in CpG sites in both FF and FFPE samples than any other CpN sites (*P* < 0.0001, One-way ANOVA with Dunnett Mulitple comparisons test). No significant difference in C > T transition at CpG or CpN sites are observed between FF and FFPE samples. **E.** C > T substitution rates in NpC sites are also comparable between FF and FFPE samples (*P* > 0.05 in all paired *t* tests). C > T substitution rate is plotted on the Y-axis and grouped according the subsequent (CpN) or antecedent base (NpC) on X-axis. All statistics are performed using two-tailed, parametric paired *t* test unless otherwise noted (GraphPad Prism Ver 6).

FFPE DNA was previously shown to contain artifacts from formalin fixation and sample preparation that result in enhanced cytosine deamination [27]. These artifacts show up as C > T or G > A (C·G > T·A) substitutions, and we expect higher C > T substitutions in the discordant positions than in concordant positions. We therefore analyze the C > T substitution rates at concordant and discordant positions. Mean substitution rates for combined C > T, G > A transitions is slightly lower in FFPE samples compared to FF sample when all SNV positions are analyzed, but the difference is not significance (Figure 6A). Interestingly, mean substitution rates for combined C > T, G > A transitions are slightly higher at discordant SNV positions, and the difference is not significant in TES data sets but significant in WXS data sets (Figure 6B). Similarly, overall mismatch error rates is slightly higher FFPE samples compared to matched FF samples, but the difference is not significant in TES data sets but significant in WXS data sets (Figure 6C). Consistent with prior studies [28–30], we found the increased rates of C > T (or G > A in complementary strand) substitutions only in the CpG context (Figure 6D) but not in other sequence contexts (Figure 6D and 6E) in both FF and FFPE samples.

### Other factors that can potentially affect concordant variant calls between FF and FFPE samples

Variant calls between matched samples may be affected by biases in coverage and mapping quality score (MAPQ) filter. Coverage in turn may affect variant allele frequency. To determine if biases in coverage could account for discordant calls between matched FF and FFPE samples, we performed coverage analysis of concordant and discordant SNV positions in WXS and TES data sets. The results indicate that the majority of discordant positions (red dots) are in low coverage regions (Figure S3).

To determine the extent to which allele fractions in variant positions affect discordant calls, we plotted the allele fraction of each variant position from the FF TES or WXS data sets on the left Y-axis and corresponding position from the FFPE TES or WXS data sets on the right Y-axis and visualized the relationship between matched samples by lines colored according to concordant (gray), discordant (red), false-positive (blue), and false-negative (green) (Figures S4). The data are also represented as the variant allele fraction correlation plot (Figures S4). In general, we did not observe an allele fraction value that could be used to filter out the majority of discordant, false-positive, or false-negative variant calls in targeted regions. However, we observed that some of the false-positive and false-negative variant calls are the results of the variant allele fraction filter (variant allele fraction >0.2 was used to make variant calls) (Figure S4). It is important to note that the majority of false-positive and false-negative calls had a variant allele fraction greater than 0, suggesting that similar variant base calls were present in both samples, although they probably did not pass the coverage or quality filter.

To determine the extent to which mapping quality scores affect discordant calls, we plotted mapping quality scores on the Y-axis (FF on the left axis and FFPE on the right axis). The relationship between two matched samples is shown by lines, color-coded as follows: concordant (gray), discordant (red), false-positive (blue), and false-negative (green). In WXS data sets, we observed that many of the false-positive and false-negative calls had a mapping quality score of <43 (Figure S5). These results are consistent with the effect of mapping quality being applied in the variant calling step. For example, in one sample a call may have be made because it passed the mapping quality threshold, but in the corresponding matched sample a call was not made, resulting in a false-negative or false-positive call. Interestingly, mapping quality of each data set was less variable within matched FF/FFPE pairs than across unrelated samples (Figure S6). These variations most likely reflect inter-operator variability in processing of biospecimen for formalin fixation and storage.

## DISCUSSION

In this study, we generated DNA sequencing libraries from a small amount of FFPE DNA. We used the DNA libraries to perform four popular Illumina sequencing approaches: whole exome sequencing, targeting sequencing, whole genome sequencing, and low-pass whole genome sequencing. DNA sequencing libraries from corresponding FF DNA were used as comparison controls.

To determine if quantitative *Alu*-based PCR is correlated with the quality of DNA, we performed quantitative Alu-based PCR. However, we did not find a correlation between *Alu*-based PCR results and library metrics (Table S1). We also ran 1 ng of starting gDNA from all FFPE samples on a high sensitivity Bioanalyzer chip (Figure S7). Some of the FFPE DNA showed broad size distribution, indicative of partial degradation of DNA. These results indicate a large variation in DNA quality from FFPE samples included in this study.

We then systematically compared concordance of reference and non-reference base calls between FF and FFPE in pair-wise comparisons in these data sets (TES, WXS, and WGS). We also performed systematic analyses of sequencing data to identify potential FFPE artifacts. In general, we observed no noticeable biases in library fragment size, coverage, or PCR duplicates between the matched FFPE and FF DNA sequencing libraries (data not shown). However, we observed small but significant increase in overall mismatch rate and C·G > T·A substitution rate at discordant SNV sites in FFPE samples in WXS data sets.

Over 99.5% concordance in base call was observed at total intersect positions in all paired samples, and over 99.9% concordance in base call was observed in targeted regions in all paired samples. In addition, 98.8% and 98.6% concordance rates in base call were observed between NGS and Omni Express Genotyping array in matched FFPE and FF samples in WGS data sets, respectively. These data suggest the robustness of Illumina sequencing for genotype calling from FFPE samples. Since FFPE DNA is susceptible to deamination and C > T substitutions [27], such artifacts may be particularly pronounced in low-input DNA library preparations, such as the approach we used in this study. The fact that we did not observe pronounced FFPE artifacts in our study may be due to relatively short storage period of FFPE blocks. Future studies that utilize much older FFPE blocks should critically evaluate newer approaches to minimize or remove FFPE artifacts, such as the treatment with uracil-DNA glycosylase or GeneRead DNA FFPE kit [27]. Additional approaches in library preparation, such as Safe-Seq [31], Dual-barcoding [32], and Circle sequencing [33] may further improve the detection of PCR artifacts and FFPE artifacts and may facilitate more accurate base calling from FFPE DNA sequencing results.

We also found high levels of concordant SNVs between FF and FFPE samples. In WXS data sets, we observed that >96% of SNVs were concordant between paired FF and FFPE samples (Figure 3A), and discordant calls were no more than 3 in exonic regions in all samples (data not shown). We did not observe any significant differences in Ts/Tv ratio between FF and FFPE samples (Figure S8). In addition, the majority of discordant calls in the WXS data sets could be effectively eliminated by using a coverage filter of 13 and higher in our data sets (Figure S9).

Our results clearly demonstrate that Illumina sequencing can be used to produce robust and reliable base calling and copy number determination from formalin-fixed tissue samples. Although our study is limited to recently archived samples, these samples may constitute translationally relevant biospecimens. For example, many ongoing clinical trials bank FFPE biospecimens for translational studies associated with clinical trials, and these samples may be ideal for next generation sequencing-based genomic studies. Moreover, it may also be possible to use older archived tissues because other studies show that SOLiD sequencing can be used to perform robust somatic mutation detections from FFPE samples that are more than 15 years old [22].

Previous studies have investigated the feasibility of next-generation sequencing from formalin-fixed samples. These studies used methods ranging from targeted sequencing of a few cancer-specific genes to whole genome sequencing. For example, Wagle *et al*. used Illumina sequencing technology and performed targeted enrichment and sequencing of 137 “actionable” genes in formalin-fixed tumor samples from 10 patients with breast or colon cancer [34]. This study reported identification of “actionable” mutations from FFPE samples that were also confirmed by a mass spectrometric-based genotyping platform.

In another study, Yost et al used SOLiD sequencing technology and performed whole genome sequencing of formalin-fixed tumor DNA from two breast cancer patients and compared the sequence results with germline DNA from fresh-frozen peripheral blood mononuclear cells (PBMC) [22]. Although the authors found increased C·G > T·A substitutions in FFPE samples compared to germline samples, they were able to apply quality filters to produce high-confidence somatic mutations in these samples. More recently, Hedegaard *et al*. compared sequencing results from matched FF/FFPE pairs and reported high levels of concordant variant discoveries between FF and FFPE samples [35]. Unlike these previous studies (Table S7), our studies demonstrate the feasibility of generating DNA sequencing libraries from a small amount of input DNA from FFPE samples and further demonstrate that these libraries can be used to perform high-pass whole genome sequencing, low-pass whole genome sequencing, whole exome sequencing, and targeted exon sequencing. These four complementary data sets will provide the research community with the ability to further explore FFPE artifacts and develop bioinformatics tools to accurately identify SNV, IDELs, and copy number alterations from FFPE DNA sequencing. Finally, we have recently performed gene expression analysis and SNV discoveries from FFPE RNA sequencing matching to the samples used in current studies. The results from these studies indicate that robust gene expression and SNV discoveries can also be made from FFPE RNA [36].

Future studies should extend our initial observations to include the effect of duration of formalin fixation and the amount of genomic DNA input on FFPE artifacts in Illumina sequencing applications. The majority of specimens used in this study underwent standard pathology processing by NCI-supported core facility, and therefore we expect less variability in processing and storage. However, research specimens with non-standardized procedure may be plagued with variable fixation times and storage, and these variabilities may need to be addressed in research-based FFPE specimens. Finally, although amount of input DNA is not an issue for bulky ovarian tumors, investigators interested in using much more limited input DNA from FFPE specimens (such as needle biopsies) should investigate the effect of input DNA amount on FFPE artifacts.

## CONCLUSIONS

In summary, our results indicate the feasibility of generating high-quality sequencing libraries and sequencing results from low input DNA extracted from formalin-fixed, archived tumor samples for targeted, whole exome, and whole genome sequencing purposes. High degree of concordant base calls at reference and non-reference positions, minimal biases in coverage, and comparable copy number alteration profiles between matched FF and FFPE in the majority of samples all suggest the potential use of FFPE samples for next-generation sequence analysis in various clinical, translational, and medical genomic studies.

## MATERIALS AND METHODS

### Sample information

Nine pairs of matched FF and FFPE samples from patients diagnosed with ovarian cancer were obtained from the Biospecimen Repository Core Facility at the University of Kansas Cancer Center. Additionally, we purchased two sample quads consisting of tumor and normal matched FF and FFPE tissues from Proteogenex, Inc. (Culver City, CA). See Table S1 for information on all samples.

All samples were collected in accordance with federal and institutional guidelines under the Institutional Review Board protocol approved by the Human Subjects Committee at the University of Kansas Medical Center. Studies were performed in compliance with the Helsinki Declaration. Fresh frozen samples were snap frozen in liquid nitrogen at the time of collection and stored at −80°C until extraction. All FFPE samples were fixed in 10% neutral buffered formalin at the time of collection (<2 days in formalin), processed by Tissue-Tek VIP for paraffin embedding, and stored at 25°C.

A significant amount of variability exists among FFPE samples depending on the fixation process, sample age, and long-term storage method [37]. Thus, it is difficult to predict how well a particular FFPE sample will perform in a sequencing assay. Although sequencing costs have dropped tremendously, it is still quite expensive and labor intensive to take a sample all the way through library prep, targeted enrichment, and sequencing to find out if it is suitable for use. To this end, we developed a QC method to assess the suitability of FFPE DNA for our Nextera-based sequencing assays. We obtained a set of 13 pairs of FFPE and matched FF samples, which varied in age, fixation method, and cancer type (Table S1). Total DNA yields from five 5-micron sections of FFPE tissue varied from 250 ng to 3 μg in 13 FFPE samples (Table S1) as a result of the differences in the tissue size in FFPE blocks.

### DNA isolation

DNA was isolated from fresh frozen tissue using the Qiagen DNeasy Blood and Tissue Kit (Qiagen, Valencia, CA) following the manufacturer's instructions. Five micron slices of FFPE specimens were dewaxed using Deparaffinization Solution (Qiagen, Valencia, CA). DNA extractions were done using Qiagen All Prep DNA/RNA FFPE Kit (Qiagen, Valencia CA), according to Qiagen's supplementary protocol for FFPE tissue (QIAamp DNA FFPE Tissue Handbook). We implemented the following protocol changes: All FFPE samples were digested with 20 μL Proteinase K shaking overnight at 56°C and then supplemented with an additional 15 μL for an additional hour for complete protein digestion. Samples were eluted in 50 μL elution buffer.

### DNA quantification and quality control

All DNA samples were quantified using Quant-iT PicoGreen dsDNA reagent according to the manufacturer's instructions (Life Technologies, Foster City, CA). One ng of each sample was run on the Agilent 2100 Bioanalyzer using the high sensitivity DNA kit (Agilent, Santa Clara, CA) to assess fragmentation and sample quality (Figure S7).

To assess the amplifiability of the DNA pairs, the following primers (Fw: 5′ GAGTTCGAGACCACCCTGGG and Rv : 5′AGAGTCTCACTCTGTAGCCCAA) were used amplify a 200 base pair fragment of a specific Alu family, AluSx_5 [38]. We chose to amplify this Alu subfamily because it is present in ∼400 copies throughout the genome, giving us a genome-wide view of amplifiability. Two ng of each DNA sample (FF and FFPE) was used per 10 μL qPCR reaction (SYBR Green Master Mix, Life Technologies) and compared to a standard curve of high quality human genomic DNA (CloneTech, Mountain View, CA). A Ct value was calculated for each sample in a FF/FFPE pair using Bio-Rad CFX software (Biorad, Hercules, CA). ΔCt values were calculated by calculating the difference between values for FFPE and corresponding FF samples (Table S1).

### Infinium genotyping assays

Infinium genotyping assays using the OmniExpress BeadChips (Illumina) were run according to the manufacturer's protocol using 250 ng of each gDNA sample unless sufficient material was unavailable. FFPE samples were restored using Infinium FFPE DNA Restoration solution (Illumina) per the manufacturer's instructions. BeadChips were scanned on an iScan and data were analyzed with GenomeStudio (Illumina) using data normalization specific for FFPE samples.

### Library preparation and targeted enrichment

All libraries were prepared from 50 ng of genomic DNA using the Nextera DNA Sample Prep Kit (Illumina, San Diego, CA) following the manufacturer's protocol. Sample specific indexes were added during 10 cycles of PCR amplification. Excess primers and primer dimers were removed using Agencourt AMPureX beads (Beckman Coulter, Danvers, MA) at a 0.8X bead ratio. Libraries were quantified by qPCR using primers specific to Illumina adaptor sequences and library size was assessed on a high sensitivity chip run on the Agilent 2100 Bioanalyer (Agilent, Santa Clara, CA).

All sample libraries underwent targeted enrichment for the whole exome, as well as a custom panel targeting ovarian cancer genes per the Nextera Enrichment Sample Prep Protocol (Illumina, San Diego, CA). Illumina's TruSeq Exome pool consisting of 90-mer biotinlyated oligos was used to enrich for the whole exome, covering known protein-coding genes, 5′ and 3′ UTRs, microRNA, and other non-coding RNA (62 Mbases total). The custom panel targeting 268 genes involved in ovarian cancer was designed and consisted of 8, 557 biotinylated probes targeting 1.7 M bases of the genome. A minimum of 500 ng of each sample library was required for enrichment, with no more than 1, 000 ng used per sample library with the exception of Samples 22285 T and 2285 N, where four samples were pooled into total enrichment. FF and FFPE paired samples were pooled at equal amounts and went through the enrichment process in the same reaction. Whole exome and ovarian targeted enrichment followed the same protocol except 5X less magnetic streptavidin beads were used to capture the pool in each hybridization step.

### Next generation sequencing

Whole genome sequencing of the single FF and FFPE matched sample pair was done on the HiSeq 2000 using 101 cycle paired end, single indexing sequencing. For whole genome sequencing, we selected sample 14119(N) because it yielded the highest amount of DNA from FFPE samples (Table S1). Whole genome sequencing produced approximately 15 billion reads for each FF and FFPE sample

Whole-exome enriched, indexed libraries were sequenced on both the HiSeq2000 and the Genome Analyzer II using 75 cycle paired end sequencing. Ovarian target enrichment samples were sequenced on the Genome Analyzer II using 75 cycle paired end sequencing. All FF/FFPE pairs were sequenced together in the same lane. For low-pass whole genome sequencing, we randomly selected 7 pairs out of 11 carcinomas.

### Sequencing data analysis

#### BWA and GATK pipeline

We used BWA (v0.6.2) aligned with seed length of 25 and default parameters, piping into Samtools (v0.1.8) to sort. Additional sorting and adding read groups were processed with Picard-tools (v1.77). All bam files were then merged with Samtools. The resulting merged file was processed with Picard-tools for PCR duplicate removal and with GATK (v2.4–7) for realignment, base recalibration, reducing of reads for faster variant calling. Consensus pileup was produced by Samtools' pileup. A hard filter (coverage >= 13 and mapping quality >= 43) was applied to consensus base call.

#### SNV calling

GATKs UnifiedGenotyper was used to call SNVs. Hard filter (coverage >= 13 and mapping quality >= 43) was applied to WGS, WXS and TEST data sets, and SNVs were annotated by customized ANNOVAR (version from Oct 2012). Coverage value was selected based on the analysis of concordant and discordant unfiltered SNV positions in WXS data sets. This analysis indicates that the majority of discordant calls have coverage of < 13 (Figure S9, denoted by a horizontal line across bar graph). Further analysis was done through several custom scripts.

#### INDEL calling

GATKs HaplotypeCaller was used to call INDELs. Recalibration was applied on WGS and WXS data, since TES call numbers were too small. Hard filters consisting of coverage (>= 13) and mapping quality (>= 43) were applied, and INDELs were annotated by customized ANNOVAR. Further analysis was done through several custom scripts. For targeted sequencing (TES), we required a minimum of 20 unique reads for variant calling since we generally obtained higher coverage in TES than in WXS.

#### CNV calling

NGSoptwin R package was used to determine the optimal window size for read count [39]. QDNAseq R package with default parameters and 15K bin size was used to produce CNV calls from low-pass whole genome sequencing data from seven FF/FFPE pairs of tissue samples [40]. CGHbase R package was used to generate frequency plot of copy number alterations [41]. CGHregions R package was used to make CNV calls of segmented region to produce the summary of copy number alterations [42].

## SUPPLEMENTARY MATERIALS FIGURES AND TABLES




