# Supplementary material for: Targeted or whole genome sequencing of formalin fixed tissue samples: potential applications in cancer genomics
Source: Oncotarget. 2015 Jul 31;6(28):25943–61. doi: 10.18632/oncotarget.4671 (PMC4694877; doi:10.18632/oncotarget.4671)
Supplement: Supplementary file 1 [file oncotarget-06-25943-s001.pdf]

SUPPLEMENTARY MATERIALS

1. Wagle N, et al. High-throughput detection of actionable genomic alterations in clinical tumor samples by targeted, massively parallel sequencing. Cancer discovery. 2:82–93. doi:10.1158/2159–8290.CD-11-0184. 2012.
2. Yost S. E, et al. Identification of high-confidence somatic mutations in whole genome sequence of formalin-fixed breast cancer specimens. Nucleic acids research. 40:e107, doi:10.1093/nar/gks299. 2012.
3. Hedegaard J, et al. Next-generation sequencing of RNA and DNA isolated from paired fresh-frozen and formalin-fixed paraffin-embedded samples of human cancer and normal tissue. PloS one. 9:e98187. doi:10.1371/journal.pone.0098187. 2014.
4. Beltran H, et al. Targeted next-generation sequencing of advanced prostate cancer identifies potential therapeutic targets and disease heterogeneity. European urology. 63:920–926, doi:10.1016/j.eururo.2012.08.053. 2013.
5. Schweiger M. R, et al. Genome-wide massively parallel sequencing of formaldehyde fixed-paraffin embedded (FFPE) tumor tissues for copy-number- and mutation-analysis. PloS one. 4:e5548, doi:10.1371/journal.pone.0005548. 2009.
6. Wood H. M, et al. Using next-generation sequencing for high resolution multiplex analysis of copy number variation from nanogram quantities of DNA from formalin-fixed paraffin-embedded specimens. Nucleic acids research. 38:e151, doi:10.1093/nar/gkq510. 2010.
7. Kerick M, et al. Targeted high throughput sequencing in clinical cancer settings: formaldehyde fixed-paraffin embedded (FFPE) tumor tissues, input amount and tumor heterogeneity. BMC medical genomics. 4:68, doi:10.1186/1755-8794-4-68. 2011.
8. Tuononen K, et al. Comparison of targeted next-generation sequencing (NGS) and real-time PCR in the detection of EGFR, KRAS, and BRAF mutations on formalin-fixed, paraffin-embedded tumor material of non-small cell lung carcinoma-superiority of NGS. Genes, chromosomes & cancer. 52:503–511, doi:10.1002/gcc.22047. 2013.
9. Spencer D. H, et al. Comparison of clinical targeted next-generation sequence data from formalin-fixed and fresh-frozen tissue specimens. The Journal of molecular diagnostics: JMD. 15623–633, doi:10.1016/j.jmoldx.2013.05.004. 2013.

SUPPLEMENTARY FIGURES AND TABLES

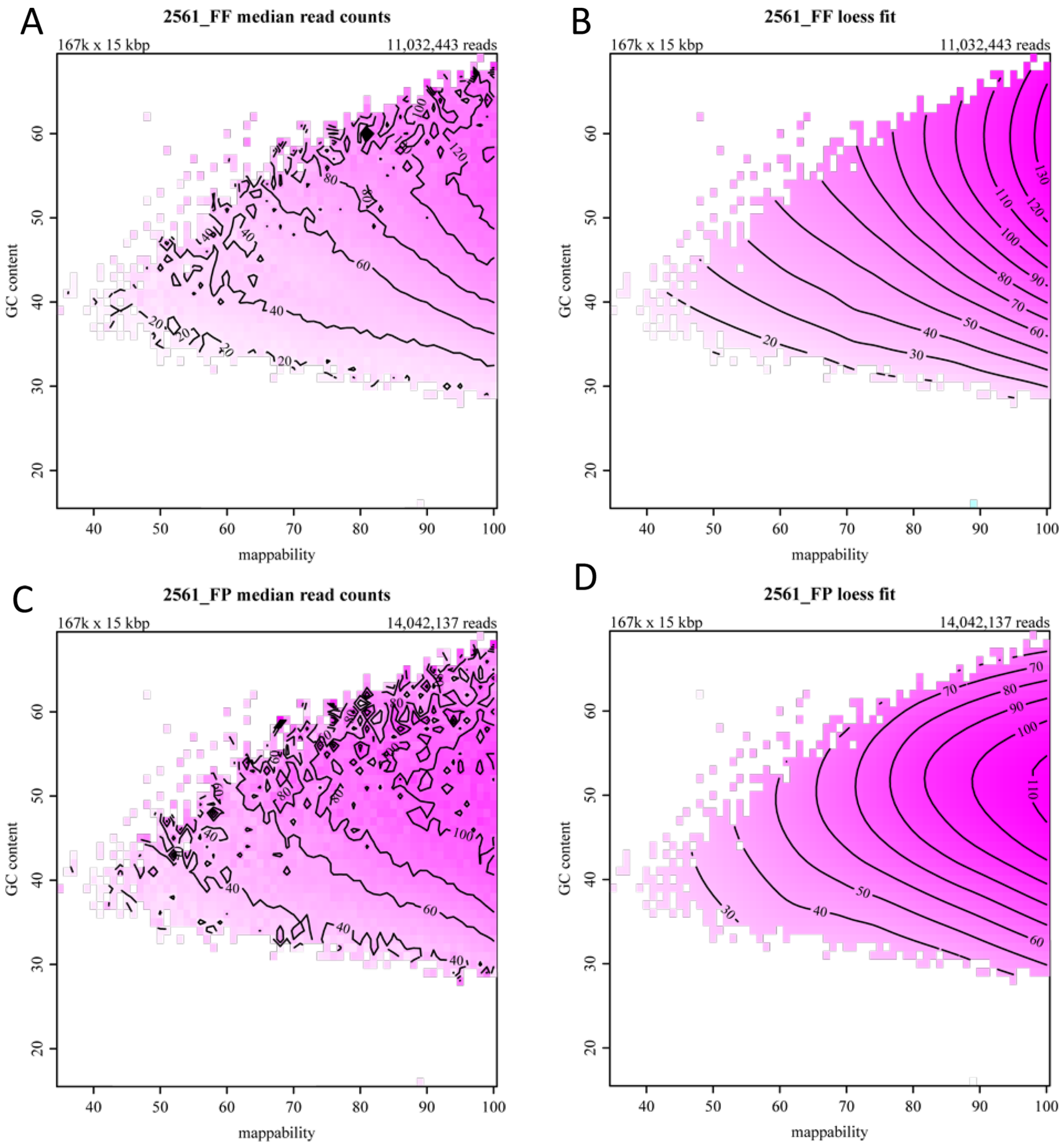

Supplementary Figure S1: Isobar plots of %GC and mappability from a representative sample set. A&C. The median read counts per bin is plotted as a function of GC content and mappability. **B&D.** In LOESS fit plots, and median read counts per bin was fitted by a LOESS surface function as described in QDNAseq R package to adjust for GC content in the bins. This procedure has been shown to stabilize the values for bins with similar GC content and mappability and produce cleaner copy number profiles.

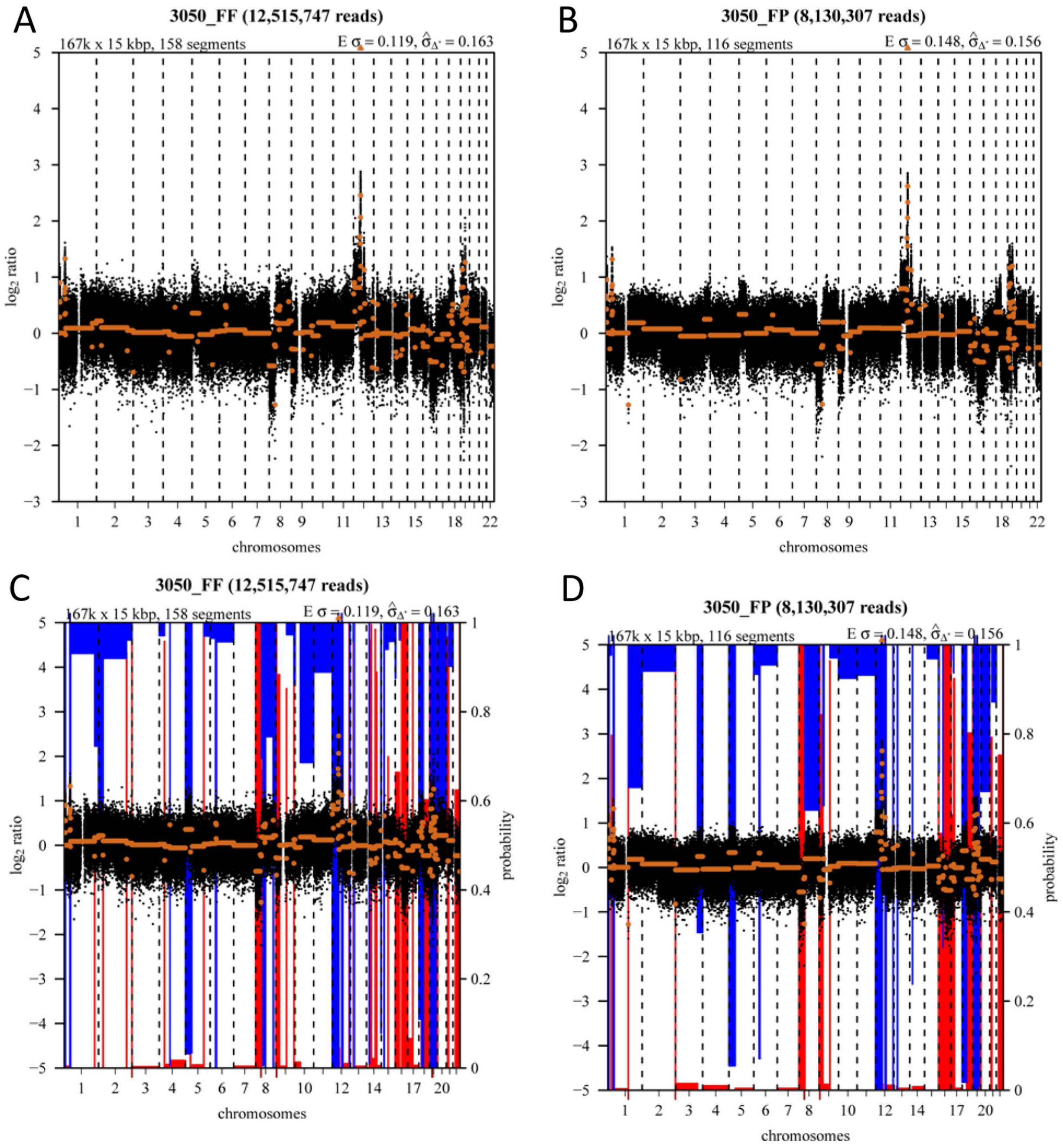

Supplementary Figure S2: Copy number profiles after GC and mappability correction from a representative sample set. QDNAseq implements several steps to eliminate spurious copy number alterations produced by problematic regions resulting from repeat regions, centromeric and telemetric regions, unresolved regions (from ENCODE list and QDNAseq list) by black listing these regions and filtering out from the data prior to CNV calling. **A–B.** Orange lines represent segmentation of normalized bins and deviation from 0 suggests potential alterations in copy numbers. **C–D.** Patient-matched fresh frozen (FF) and formalin-fixed paraffin-embedded (FP) show similar profiles. Copy number gain and loss calls were made using QDNAseq and indicated by blue (gain) and red (loss) bars, and the height of the colored bars indicate the probability of CNV calls.

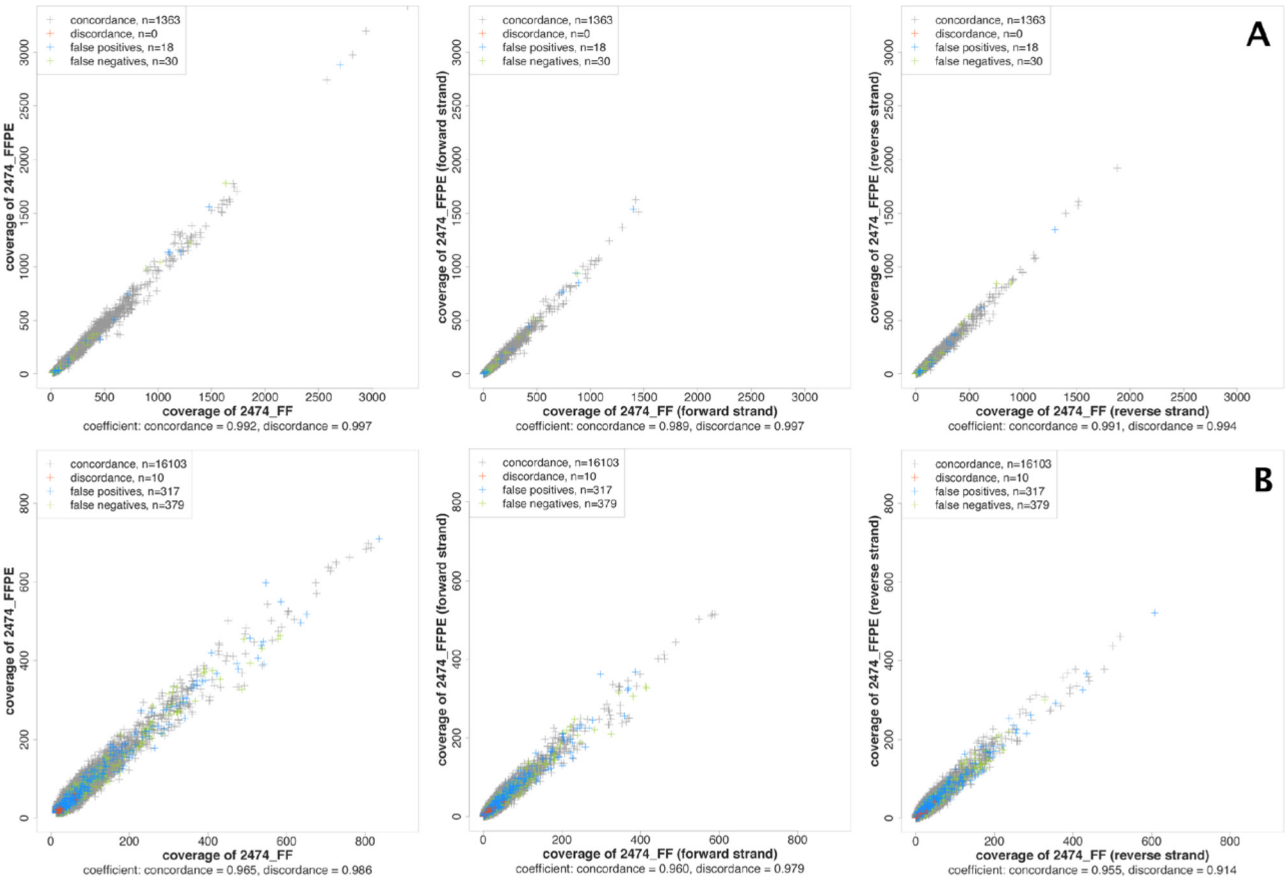

Supplementary Figure S3: Coverage correlation of non-reference (variant) positions. One representative of TES in targeted regions **A.** and of WXS in exonic regions **B.** are shown. Coverage (total reads, left panel; forward strands only, middle panel; and reverse strand only, right panel) for each genomic position with variant calls are plotted for FF (x-axis) and FFPE (y-axis). The majority of points fall in the line x = y, indicating minimal coverage bias between FF and FFPE for these positions. Discordant calls, indicated by red, are mainly associated with low coverage positions. False positive calls (variant calls made in FFPE but not in matched FF DNA) are shown in blue, and false negative calls (variant calls made in FF but not in matched FFPE DNA) are shown in green. These results indicate that low coverage contributes substantially to discordant calls but not to false-positive and false-negative calls.

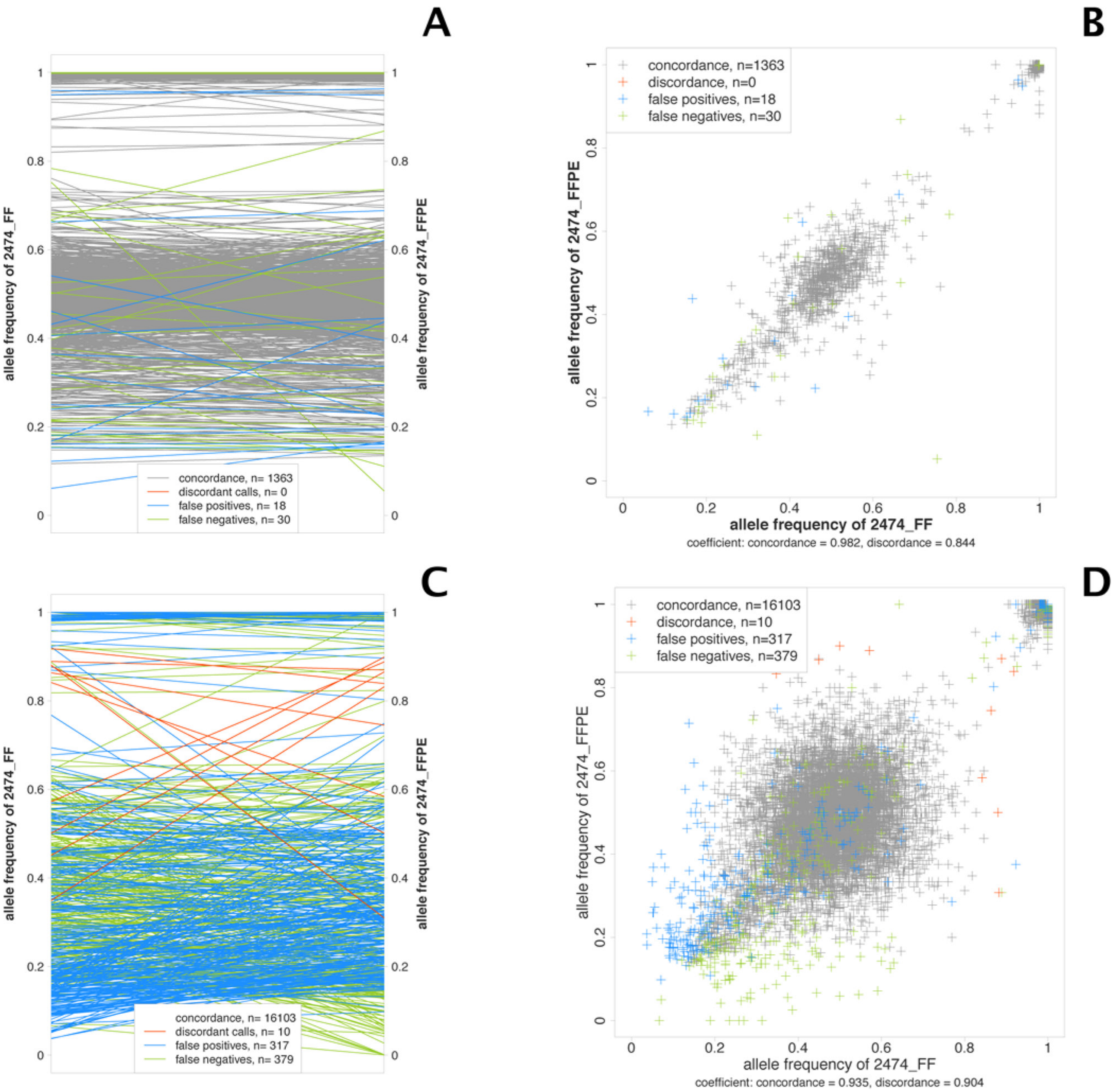

Supplementary Figure S4: Allele frequency of non-reference positions. Allele frequencies of non-reference (variant) positions in targeted regions of TES **A&B.** and exonic regions of WXS **C&D.** are plotted for a representative paired samples. A&C: Allele frequencies of variant calls in FF (left y-axis) and FFPE (right y-axis) are connected by lines to indicate their relationships in the plots. B&D: Allele frequencies of variant calls in FF (x-axis) and FFPE (y-axis) are plotted. Discordant calls are indicated by red lines or crosses. False positives (variant calls made in FFPE but not in FF) and false negatives (variant calls made in FF but not in FFPE) are indicated by blue and green lines or crosses, respectively. These results indicate that differences in allele frequency of variant calls between FF and FFPE contribute to false-positive and false-negative calls.

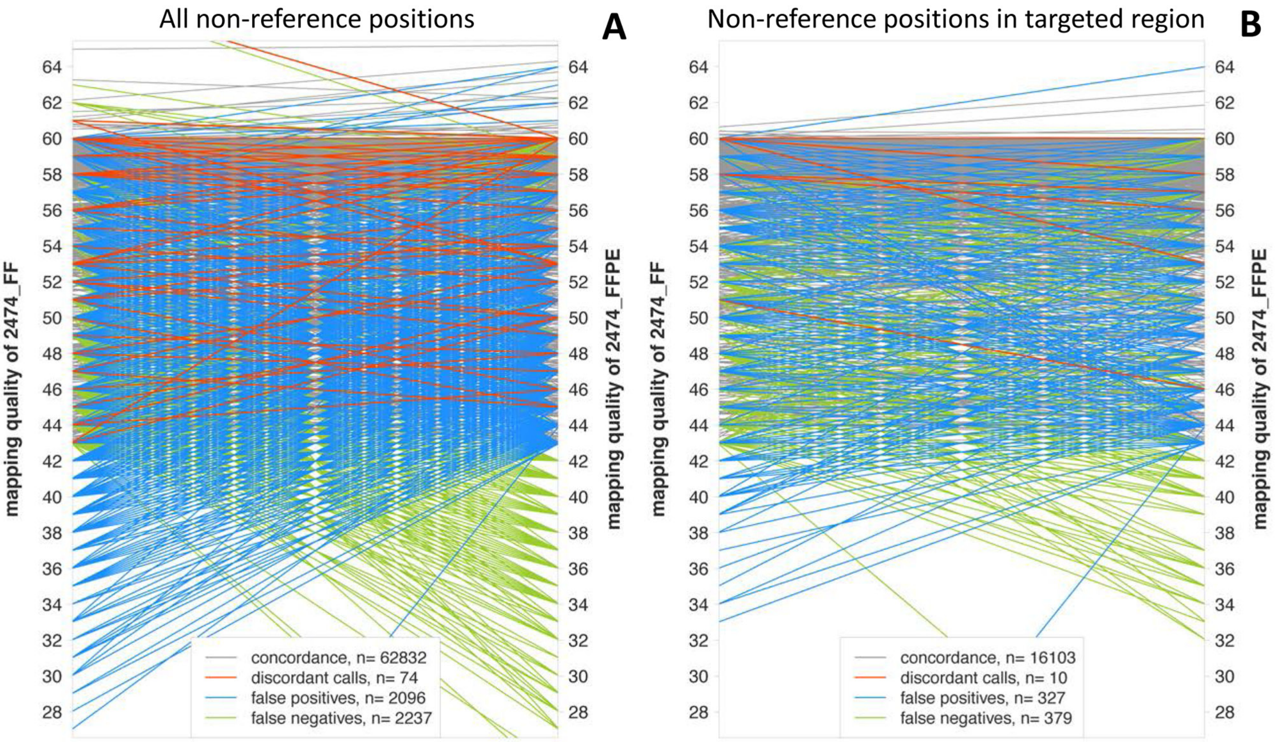

Supplementary Figure S5: Mapping quality of non-reference positions from total and targeted regions. Total **A.** and targeted **B.** regions from a representative of WXS data sets are shown. Mapping quality score of variant position in FF (left y-axis) and FFPE (right y-axis) are plotted and connected by lines to indicate the relationship. The results indicate that differences in mapping quality contribute to some of the false-positive or false-negative variant calls in the data sets.

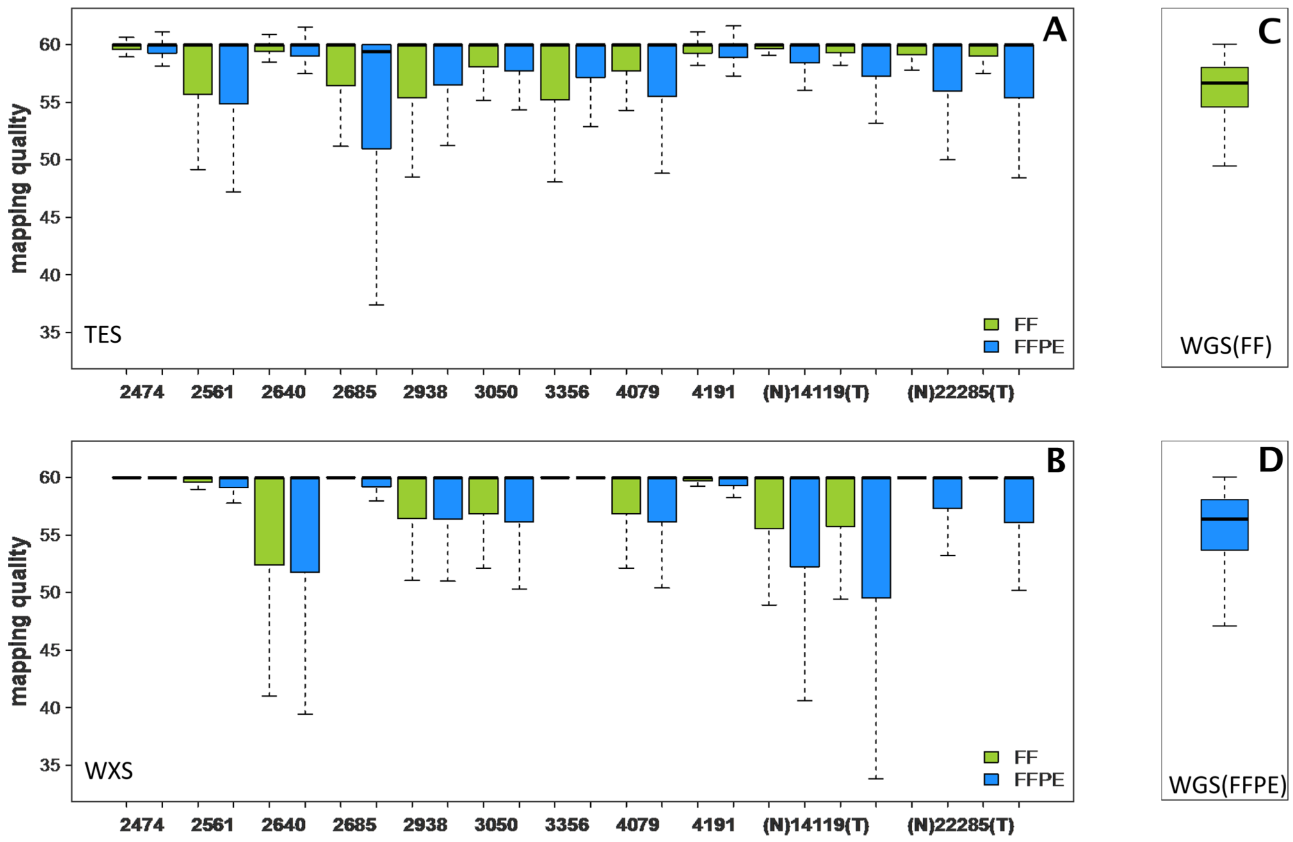

Supplementary Figure S6: Variations in mapping quality across samples. Mapping quality boxplot without outliers from total positions in TES **A.** WXS **B.** WGS FF **C.** and FFPE **D.** respectively. The results indicate minimal variability in mapping quality within paired FFPE/FF samples but larger variability among patient samples.

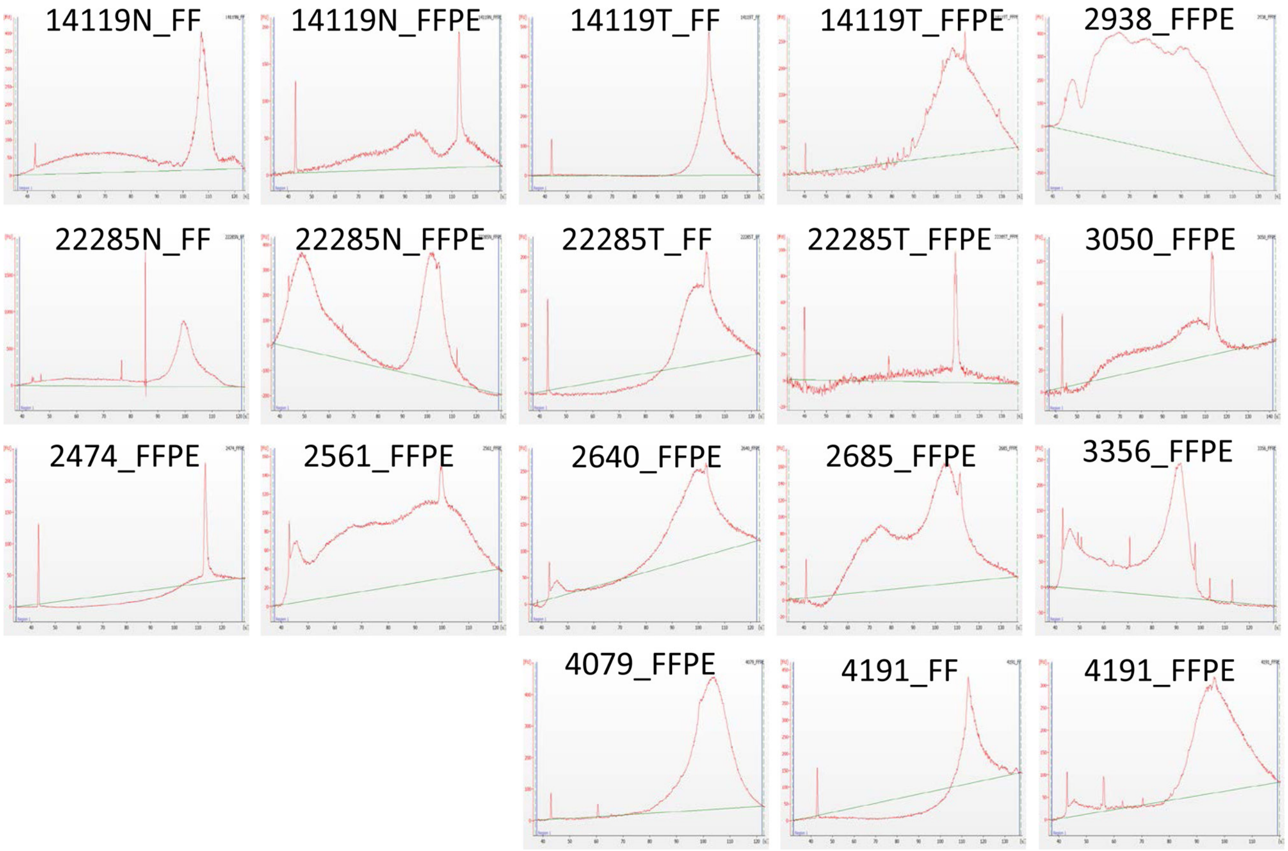

Supplementary Figure S7: FFPE QC Metrics as determined by Agilent Bioanalyzer. Some of the FFPE samples showed a broad shoulder or peaks at low molecular weight, indicative of DNA degradation.

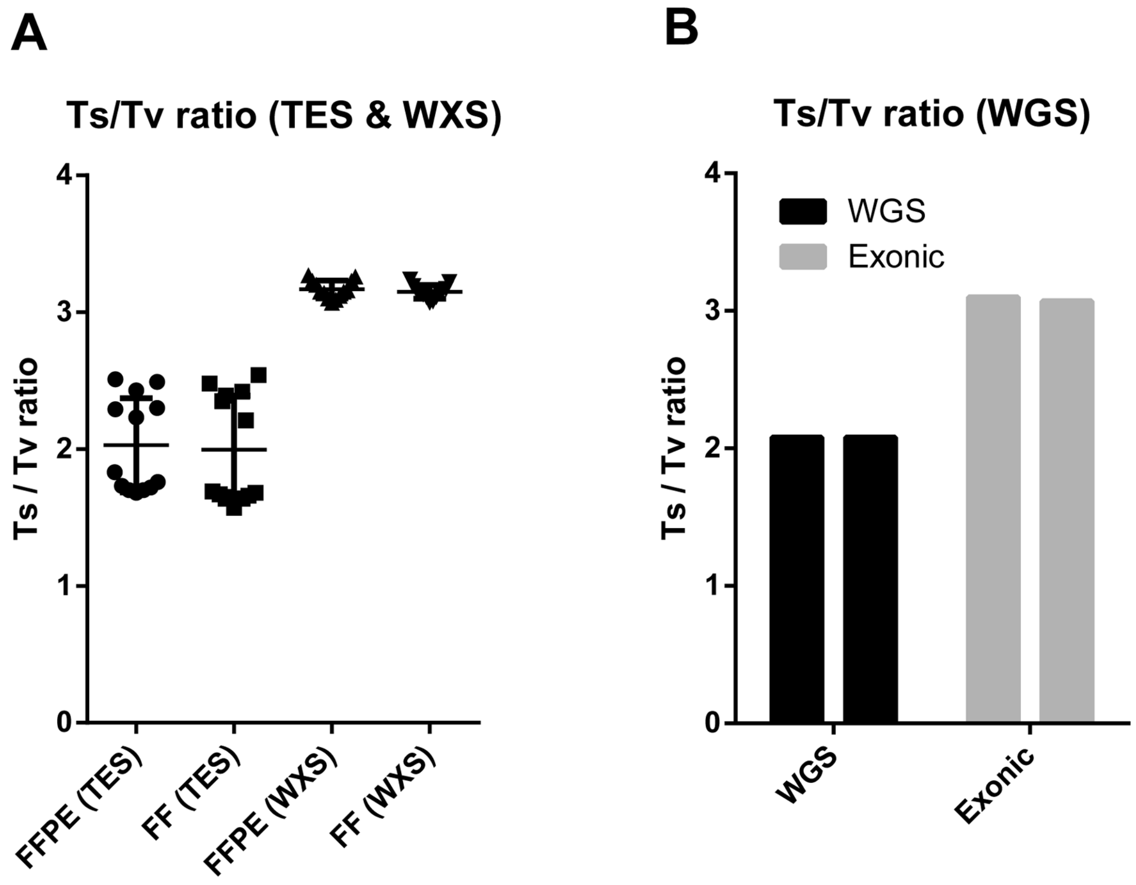

Supplementary Figure S8: Comparison of Ts/Tv ratios between FF and FFPE samples from TES, WXS, And WGS data sets.

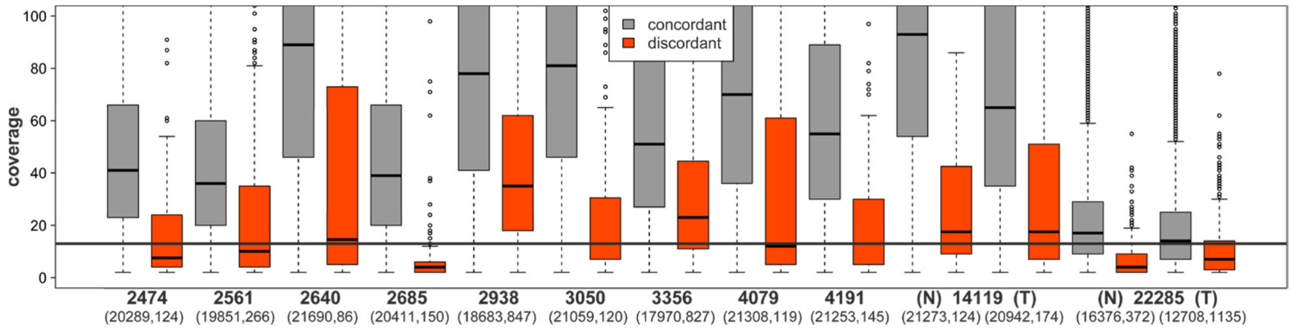

Supplementary Figure S9: Coverage distribution of concordant and discordant non-reference positions in WXS data sets. Number of data points for each group are shown in parenthesis with concordant followed by discordant data points.

Supplementary Table S1. Clinical information for FF/FFPE samples used in this study
AluQC PCR was performed to assess amplification efficiency of human Alu-sequences. Amplification efficiency should correlate well with the quality of DNA. % of FF Library size indicate the library size of FFPE relative to corresponding FF library. % PCR duplicates were determined by Picard tools. Variability in DNA yield is likely due to differences in the size of embedded tumor samples.

Supplementary Table S2. Concordance of base calls between FF and FFPE samples.

Supplementary Table S3. Concordance of SNV cslls between FF and FFPE samples.

Supplementary Table S4. Concordance of InDel calls between FF and FFPE samples.

Supplementary Table S5. Low-pass whole genome sequencing of FF and FFPE tumor pairs
Millions of 35-bp reads (M), percentage of mapped reads and number and percentage of reads with mapping quality score >=30 are shown.

Supplementary Table S6. Summary statistics of CNV regions between FF and FFPE samples
Copy number variant regions, generated by QDNAseq and summarized by CGHregions, are described as the summary statistics. The median sizes of copy number gain and loss regions between FF and FFPE groups are comparable. Total numbers of events in each group are also listed.

Supplementary Table S7. Methods and analyses in prior studies compared to current study

Study	FFPE	Matched FF	Methods	Platform	Analysis
Wagle et al.[1]	10 tumors		137-genes targeted sequencing	Ilumina	SNVs, CNVsMass-spectrometric genotype verification
Yost et al.[2]	2 samples	PBMC (fresh frozen)	Whole genome sequencing	SOLiD	SNVsFFPE artifacts
Hedegaard et al.[3]	36 tumors	36 tumor13 normal	Whole exome sequencingRNA sequencing	Illumina	SNVs and gene expression; analysis with CLC Bio
Beltran et al.[4]	50 tumors	No matched FF	182 cancer-associated genes; 37 introns; 14 rearranged genes	Illumina	Low input DNASNVs and CNAsFISH verification
Schweiger et al.[5]	3 samples(2 normal, 1 tumor)	3 samples	Low-pass whole genome sequencing	Illumina	CNVs
Wood et al.[6]	1 cell line2 tumors	3 samples	Low-pass whole genome sequencing	Illumina	CNVs
Kerick et al.[7]	5 tumors	5 tumors	Whole exome sequencing	Illumina	SNVs, InDels, CNVs
Tuononen et al.[8]	81 tumors	No matched FF	192-genes targeted sequencing	Illumina	SNVsPCR verification
Spencer et al.[9]	16 tumors	16 tumors	27 cancer-related genes	Illumina	SNVs, SNP array verification
Current study	11 tumors2 normal	11 tumors2 normal	Whole exome sequencing, targeted sequencing, whole genome sequencing, Low-pass whole genome sequencing	Illumina	SNVs, InDels, CNVsVerification with SNP array for base calls; low input DNAFFPE artifacts; analysis with open-source bioinformatics
